# Open Window Class Rooms

**Published:** 1913-11

**Authors:** Walter W. Roach

**Affiliations:** Supervisor of School Medical Inspectors, 4th and 5th Districts, Philadelphia


### Open Window Class Rooms

BY DR. WALTER W. ROACH
Supervisor of School Medical Inspectors, 4th and 5th Districts, Philadelphia

IF in open window class-rooms we have a means of fortifying the child against disease, and in fresh, cold air a mental stimulant that can be controlled so as not to cause overstrain, then here is an agency for teaching to parents through the child the benefits of ventilation and hygienic surroundings which will aid materially in repelling the spread of tuberculosis in the community.

It is important, therefore, that the employment of lower temperature methods should be supervised by medical authority and not incautiously applied by laymen (teachers, etc.) in a way to do quite as much harm as good, no matter how sincere the good intent.

Care should be observed that the children in open window and fresh air class-rooms are provided with sufficient outer garments to retain the body generated heat. The effect of cold air is to create a desire for exercise—a physiologic demand for active circulation. Therefore, proper exercise should be given at frequent intervals between lesson periods, but never prolonged to fatigue, nor violent enough to excite perspiration. A soft woolen blanket should be wrapped around the feet and legs, and hood or cap, mittens and sweaters worn. This will maintain equilibrium of circulation and keep the child warm.

It should be remembered that cold vitiated air is as harmful as warm impure air. The virtue is in the purity of the air and not alone in its low temperature.

---

HYPERTHERMIC TREATMENT OF GONORRHOEA. Majors L. W. Harrison and G. J. Houghton, *Jour. of the Royal Army Med. Corps,* February, 1913, state that the gonococcus cannot resist a temperature of 104 F. for six hours, while the urethra can stand a temperature of 114-119 or even 122. They call attention to a fact often observed by clinicians but little known in literature, that gonorrhœa frequently subsides spontaneously during high fever or other cause, as typhoid. They apply heat to the urethra by a double catheter, the outer closed except for an outlet near the external end, the inner tube carrying a current of hot water. Treatments occupy half an hour. They report sixteen cases, successfully treated in 4-11 days, without complications.

